# Endobronchial Ultrasound-Guided Transbronchial Needle Aspiration (EBUS-TBNA) in Moderate Sedation in Patients Aged 80 Years and Older

**DOI:** 10.3390/jcm15114084

**Published:** 2026-05-25

**Authors:** Eleonora Casalini, Matteo Fontana, Roberto Piro, Patrizia Ruggiero, Sofia Taddei, Luca Ronzoni, Laura Rossi, Nicola Facciolongo

**Affiliations:** Pulmonology Unit, Azienda Unità Sanitaria Locale-IRCCS di Reggio Emilia, 42122 Reggio Emilia, Italy

**Keywords:** endobronchial ultrasound, transbronchial needle aspiration, elderly, complication, safety

## Abstract

**Background/Objectives**: The utility and safety of EBUS-TBNA in the general population are well-documented in the scientific literature, but data on elderly patients, particularly those over 80 years old, are limited. **Methods**: We retrospectively investigated patients aged 80 and over who underwent EBUS-TBNA under moderate sedation, without anesthesiologic assistance, over a period of seven years at a single Italian hospital. The primary outcome was the safety of the procedure assessed by evaluating the complication rate occurring during the procedure and up to one week afterward. Secondary outcomes included the rate of procedures with successful sampling, the percentage of procedure repeated with anesthesiologic assistance, specimen adequacy, overall diagnostic yield, type of diagnosis, malignancy rate and the percentage of patients who underwent active treatment after obtaining an oncological diagnosis. **Results**: A total of 141 patients were enrolled in the study; the mean age was 82.6 ± 2.2 years. The incidence of complications was 11.3% (16/141); most adverse events were mild (9.9% 14/141). Two patients (1.4%) experienced major complications requiring hospitalization. Regarding the secondary endpoints, sampling was successfully performed in 99.3% of cases (140/141); in one case, it was necessary to repeat the procedure with anesthesiologic assistance (0.7% 1/141). Specimens were adequate in 96.4% of cases (135/140); the overall diagnostic rate was 85.7% (120/140); the malignancy rate was 52.9% (74/140) and among these, 78.4% (58/74) were treated. **Conclusions**: EBUS-TBNA performed under moderate sedation is a safe and diagnostically effective procedure in patients older than 80 years of age, supporting its use in this growing population.

## 1. Introduction

Endobronchial ultrasound-guided transbronchial needle aspiration (EBUS-TBNA) is a minimally invasive endoscopic procedure widely used in interventional pulmonology for the diagnosis of mediastinal adenopathy or lesions adjacent to the central airway [1,2], as well as for lung cancer diagnosis and staging [3,4].

Life expectancy has significantly increased in recent decades particularly in high-income countries, where nearly half of the global disease burden occurs in older adults. Malignant neoplasms, chronic respiratory diseases and respiratory infections are among the leading contributors to disease burden. For this reason, diagnostic procedures are frequently required in older patients in order to guide appropriate therapeutic strategies [5].

Although the utility and safety of EBUS-TBNA in general population are largely documented in the scientific literature, data on elderly patients, particularly those over 80 years old, remain limited, with only a few studies specifically addressing this subgroup.

Some reports suggest a higher complication rate in individuals aged 80 and older who undergo bronchoscopy [6,7]; additionally, the American College of Chest Physicians Quality Improvement Registry Evaluation and Education (AQuIRE) Registry reported a more frequent escalation in the level of care due to complications in patients aged > 70 years who underwent EBUS-TBNA [8].

For these reasons, establishing whether EBUS-TBNA is safe in this patient group is highly relevant to ensure a safe diagnostic procedure and potentially offer therapeutic options.

EBUS-TBNA may be performed under general anesthesia (via endotracheal tube, laryngeal mask, or rigid bronchoscope), or under moderate sedation trough the mouth or nose, with pharmacologically induced depression of the level of consciousness. Previous studies have demonstrated no significant difference in diagnostic yield and sensitivity between EBUS-TBNA performed under general anesthesia and that conducted in moderate sedation [9,10,11,12]. The choice of sedation is largely influenced by institutional resources and clinical practice; moderate sedation allows for the procedure to be performed even in centers without routine anesthesiologic support for endoscopic procedure, thereby improving accessibility and procedural flexibility.

The aim of this study is to evaluate safety and efficacy of EBUS-TBNA performed under moderate sedation without anesthesiology assistance in patients aged 80 years and older.

## 2. Materials and Methods

This is a single-center retrospective study including consecutive patients aged ≥ 80 years and older who underwent EBUS-TBNA during a 7-year period from 1 January 2017 to 31 December 2023 at the Interventional Pulmonology Unit of Reggio Emilia, a tertiary referral center in Italy.

The study was approved on 23 July 2024 by the Institutional Review Board of the “Area Vasta Emilia Nord” (229/2024/OSS/AUSLRE) and was conducted in accordance with the World Medical Association Declaration of Helsinki. Written informed consent was obtained from all patients before EBUS-TBNA was performed; whenever possible, written informed consent for study participation was also collected. In consideration of the features of the retrospective study, the Institutional Review Board authorized the analysis of the data related to the patient who was not able to be reached to ask for consent. Privacy and anonymity were ensured for unreachable patients. The study was performed using the electronic database that collects all data on interventional procedures conducted at our hospital.

Consecutive subjects aged 80 years and older who underwent EBUS-TBNA in moderate sedation without anesthesiology assistance during the study period were enrolled.

Indications for EBUS-TBNA procedure included the presence of radiological enlargement of mediastinal lymph nodes (short-axis > 1 cm) and/or increased FDG activity on FDG-PET/CT, diagnosis of PET-positive lesions adjacent to the central airway, and mediastinal nodal staging.

EBUS-TBNA procedures performed under general anesthesia or deep sedation using laryngeal mask or rigid bronchoscope were excluded from the analysis, as intubated or tracheotomized patients. Subjects presenting with severe hemodynamic instability and an American Society of Anesthesiologists (ASA) score > of 3 were not considered clinically eligible for the procedure under moderate sedation; subjects taking anticoagulant or antiplatelet agents (other than aspirin) had to withhold the medication according to the standard recommended period; if this interruption was judged clinically contraindicated, the patient was ruled out of the study. Patients who underwent others diagnostic procedures in the same session (such as bronchoalveolar lavage or transbronchial biopsy) were excluded.

Before the EBUS-TBNA procedure, all subjects underwent a detailed clinical evaluation, laboratory tests, and imaging assessment with computed tomography (CT) and positron emission tomography (FDG-PET).

All interventional pulmonology procedures considered in the study were performed in accordance with international recommendations [13] through oral or nasal routes [14] with an EBUS bronchoscope that has an outer diameter of 6.9 mm (Olympus BF-UC180F, Tokyo, Japan) or 6.6 mm (Olympus BF-UC190F). EBUS-TBNA procedures were performed by experienced interventional pulmonologists supported by two nurses specialized in this field. In some cases, a balloon device (Olympus, Japan) filled with sterile saline was positioned to obtain a better ultrasound image.

Sedation protocols and drug dosages were tailored to individual patient characteristics by the bronchoscopist, following current clinical practice for moderate sedation. All procedures were performed under moderate sedation using intravenous (IV) midazolam, supplemented with either meperidine or fentanyl. Midazolam (0.01–0.1 mg/kg) was diluted to 1 mg/mL in normal saline and administered in 1–2 mg boluses. When used, fentanyl (25–200 μg total) was diluted to 10 μg/mL and administered in 25–50 μg boluses. Meperidine (1 mg/kg) was diluted to 10 mg/mL and administered prior to the procedure. Dosages were titrated at the discretion of the bronchoscopist to achieve the desired depth of sedation while ensuring patient safety.

All patients received local anesthesia in each nostril, in the oropharynx, in the larynx, and in the lower airways. The lowest dose of lidocaine necessary to ensure adequate bronchoscopic conditions and patient comfort was used; approximately 4–6 mg/kg of lidocaine were administered.

Patients received oxygen through a nasal cannula to keep the SpO_2_ above 90% and underwent continuous multiparameter monitoring (saturation, electrocardiogram, and regular blood pressure measurements). Usually in the same session, patients undergo a flexible bronchoscopy to examine the tracheobronchial tree before the EBUS-TBNA procedure; explorative bronchoscopy was avoided only if it had already been performed in the days before.

Subjects taking anticoagulant or antiplatelet agents (other than aspirin) had to withhold from taking the medication, according to the standard recommended period [15,16].

Usually, during EBUS-TBNA procedures, at least three passes are performed at each lymph node station; after removing the stylet, the specimen was collected by aspiration using a syringe, while the needle was moved back and forth approximately 10–20 times within the lesion. From aspirates, a cell block for histological analysis and slides fixed in 95% alcohol for cytological examination were obtained; in some cases, rapid on-site evaluation (ROSE) was performed, depending on the availability of a second pulmonologist or a biologist trained in this technique. The procedure planning (the location and number of lymph node stations to sample) was previously determined case by case by the bronchoscopist, based on the imaging and the diagnostic purposes for each patient.

After the procedure, outpatients were monitored for a period of at least two hours and then discharged, while inpatients returned to their respective hospital wards and were monitored for the next two hours by the staff of the referring department.

If complications occurred during hospital observation, the bronchoscopist was consulted and adverse events were recorded; in cases of late complications, patients were usually referred to the emergency department and complications were identified through a review of any hospitalizations or emergency department visits in the week following the bronchoscopy. All data was collected from the electronic medical records.

The primary outcome of the study was to assess the safety of EBUS-TBNA in moderate sedation in patients aged 80 years and older, evaluating the overall complication rate occurring during the procedure and up to 1 week after the procedure. According to the British Thoracic Society guidelines, major complications include severe bleeding (defined as bleeding requiring the placement of a bronchial blocker, blood transfusion, admission to critical care unit or death), cardiac arrhythmia requiring treatment, seizures, myocardial infarction/pulmonary edema, pneumothorax requiring aspiration/intercostal drain, oversedation requiring ventilatory support or reversal, hospitalization, admission to intensive care unit, or death. Minor complications include transient hypoxemia, mild bleeding (defined as bleeding stopping spontaneously or requiring continued suctioning of blood from the airways) or moderate bleeding (bleeding requiring intubation of the biopsied segment with the bronchoscope in wedge position or the use of adrenaline or cold saline to stop the bleeding); cardiac arrhythmia not requiring treatment, hypertension or hypotension, transient bronchospasm/laryngospasm, fever, infections, or poor tolerance for excessive cough with early interruption of the procedure [17].

Secondary outcomes include the analysis of performance elements, evaluated at both the patient and procedure levels.

The following variables were assessed for each patient: demographic information including age, gender, body mass index (BMI), comorbidities and the Charlson Comorbidity Index (CCI); ASA score; survival status at the time of data collection; indication for EBUS-TBNA; the amount of sedatives used; and percentage of patients undergoing an active treatment after obtaining an oncological diagnosis through the endoscopic procedure.

The following variables were assessed for each procedure: percentage of procedure in which sampling was performed, percentage of procedure repeated with anesthesiologic assistance, and nodal stations sampled based on the International Association for the Study of Lung Cancer (IASLC) classification [18]; number of stations sampled per procedure and number of passes for each lymph node station; adequacy of specimens, defined as samples either revealing a definitive diagnosis or showing a preponderance of lymphocytes; if no diagnoses were made and insufficient lymphocytes were available, the samples were considered inadequate; overall diagnostic yield, defined as cytopathological findings revealing a clear diagnosis such as a malignant disorder, a granulomatous disorder (sarcoidosis or tuberculosis) or other conditions such as anthracosis; and type of diagnosis and malignancy rate, defined as the rate of malignant disease diagnosis.

Statistical analysis was performed with the software GraphPad Prism version 9 for MacOS (GraphPad Software, San Diego, CA, USA, www.graphpad.com). Continuous variables are presented as mean and standard deviation, while categorical variables are expressed as absolute values and percentages. Survival analysis was performed using Kaplan–Meier survival function estimates, and the difference between survival functions was compared with the log rank test. A multivariate analysis using Cox regression was performed to identify factors associated with survival. A multiple logistic regression with the occurrence of complication as the dependent variable was also performed. A *p*-value < 0.05 was considered statistically significant.

## 3. Results

**Figure 1:**
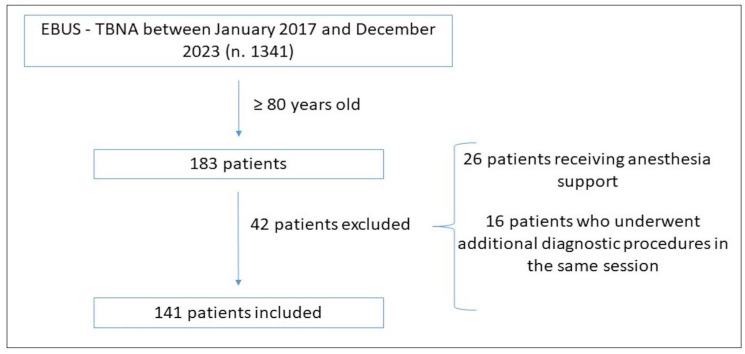
Enrolment process diagram.

Between 1 January 2017 and 31 December 2023, 183 patients aged 80 years and older underwent a linear EBUS-TBNA at our center; among these, 141 patients (84 men and 57 women) underwent linear EBUS-TBNA under moderate sedation without anesthesiological assistance and without other diagnostic procedures in the same session (such as bronchoalveolar lavage or transbronchial biopsy) (Figure 1).

Mean age was 82.6 ± 2.2 years, the oldest patient was 90 years old, the mean CCI was 5.2 ± 0.9, and the mean ASA score was 2.6 ± 0.6; the demographic characteristics of the study patients are summarized in Table 1.

The main indication for EBUS-TBNA procedure was the diagnosis of mediastinal lymph nodes or lesions adjacent to the central airway (97 patients, 68.8%); mediastinal nodal staging was performed in 44 patients (31.2%). The oral and nasal route were used in 51.1% and 48.9% of cases, respectively.

In most cases, TBNA sampling was performed (99.3%, 140/141); it was not possible to carry out the sampling (0.7%, 1/141) only in one case due to severe patient agitation, and the procedure had to be repeated with anesthesiological assistance. Multiple lesions were punctured in 65 cases (46.1%) and the median number of punctures for each station was 4.1 (range 1–7). The mean sedative doses were midazolam 4.6 ± 2 mg, fentanyl 0.1 ± 0.09 mg and meperidine 72.4 ± 12.4 mg. Sampling information and the main doses of sedation are summarized in Table 2 and Table 3, respectively.

Specimens were adequate in 96.4% of cases (135/140 samples); among these, in 10.7% of cases (15/140), the sample showed a preponderance of lymphocytes without any other specific feature; sampling outcomes are summarized in Table 4. The sample was inadequate in five patients (3.6%); in all of them, another diagnostic procedure was planned.

The overall diagnostic yield was 85.7% (120/140); the malignancy rate was 52.9% of cases (74/140). Specifically, non-small cell lung cancer (NSCLC) was the most common diagnosis (34.3% of patients), while small cell lung cancer (SCLC) was observed in 10% of patients, and lymphoma in 2.85% and metastasis in 5.7% of patients; anthracosis was observed in 30% of cases and granulomatous reaction in 2.85% of cases.

Among oncological patients, 58 subjects (58/74, 78.4%) received active treatment; 29 patients underwent systemic oncological therapy (29/58, 39.2%), 20 underwent radiotherapy (20/58, 27%) and nine patients underwent a combination of both (9/58, 12.2%); nobody underwent surgery treatment after an oncological diagnosis; the data are summarized in Table 5. Sixteen (16/74, 21.6%) patients underwent best supportive care due to poor general condition.

A total of 125 of patients (88.6%) had no complications; overall, 16 complications were recorded (11.3% of all procedures), as summarized in Table 6, and most of them were minor complications (14 cases, 9.9% of all the procedures); the most frequent adverse event was blood pressure elevation requiring the administration of intravenous antihypertensive drugs (4.3%); three patients (2.1%) presented with transient hypoxemia requiring high-flow oxygen administration; two patients (1.4%) experienced mild epistaxis, and in one case (0.7%), a minor bleeding occurred after the first needle puncture and was controlled with continued suctioning. One patient (0.7%) presented with a cough and poor tolerance to the procedure, while in one case (0.7%), transient laryngospasm without hypoxemia occurred. Two patients (1.4%) experienced major complications necessitating hospitalization. The first patient developed pneumothorax, requiring chest-tube drainage, while the other presented with epistaxis requiring tampon placement during the observation period after the procedure. Successive blood tests detected thrombocytopenia necessitating platelets transfusion; in the following days, the patients presented with hematemesis with severe anemia, and died a few days later due to persistent thrombocytopenia, the onset of respiratory failure with evidence of a lymphangitic pattern at CT scan, and a rapid deterioration of general condition. No infectious complication or severe bleeding were recorded.

Overall, eight patients (5.7%) died within one month and 18 (12.8%) within three months after EBUS-TBNA. The deaths were due to the rapid deterioration of clinical condition due to the progression of the underlying diseases and were not related to the endoscopic procedure (Table 7).

**Figure 2:**
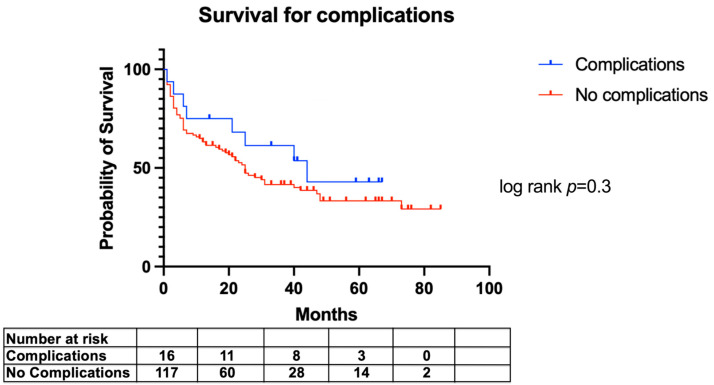
Survival for complications.

**Figure 3:**
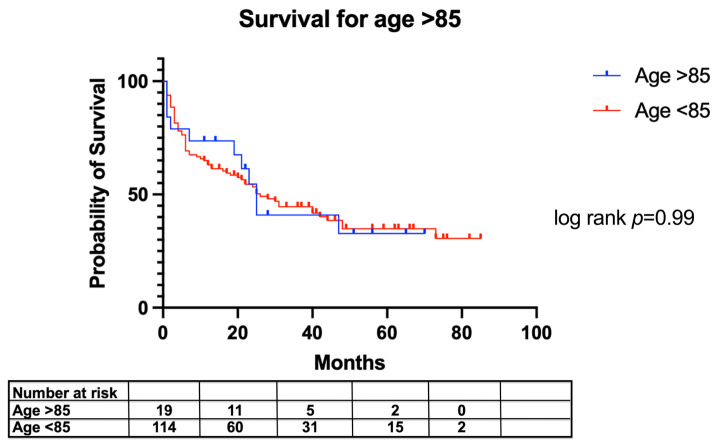
Survival for age > 85.

**Figure 4:**
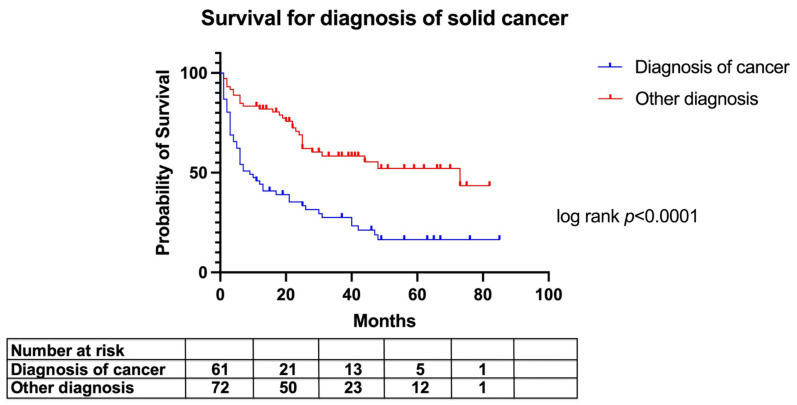
Survival for diagnosis of solid cancer.

In order to better evaluate the impact of the procedure and its complications on our cohort of patients, we built a multivariate logistic regression model showing that the occurrence of complications was not associated with age (OR = 0.89; 95% CI: 0.64–1.16; *p* = 0.43), sex (OR = 0.67; 95% CI: 0.21–2.15; *p* = 0.49), BMI (OR = 1.04; 95% CI: 0.89–1.21; *p* = 0.61) and CCI (OR = 0.58; 95% CI: 0.39–1.49; *p* = 0.46). Furthermore, a Cox regression analysis was performed, showing that the only factor associated with a prognostic impact was the presence of a diagnosis of a solid cancer (hazard ratio 3.136, 95% CI 1919–5211). As shown with the Kaplan–Meier survival analysis in Figure 2, Figure 3 and Figure 4 respectively, there was no difference in survival between those who experienced complications and those who did not, nor between patients older or younger than 85 years, but there was a significant difference depending on the presence of a diagnosis of solid cancer (*p* < 0.0001). The mean follow up was 24.5 ± 22.9 months.

## 4. Discussion

Utility and safety of EBUS-TBNA in the general population are well-documented in the scientific literature, but data in elderly patients remain limited. Some studies have reported a higher complication rate in individuals aged 80 years and older who underwent bronchoscopy. Rokach et al. analyzed bronchoscopy performed in 150 patients aged 80 years or older, including patients on mechanical ventilation; in this study, the frequency of complications (11.5%) and the mortality rate (1.2%) were significantly higher in octogenarians compared with the control group, with no difference in the frequency of complications between ventilated and non-ventilated patients [7]. Haga et al. evaluated bronchoscopy in a prospective study including 66 patients aged 80 years and older; the rate of complications was higher in very elderly adults than in the younger patients, although the difference was not statistically significant. The authors concluded that bronchoscopy is not as safe in very elderly adults as it is in younger adults and that the indication for the exam should be carefully considered in this group of individuals [6].

A certain number of studies have investigated the safety of EBUS-TBNA under moderate sedation in elderly patients, especially in people aged 65 years or older, showing positive results; however, very few studies have specifically addressed patients aged 80 years or older. To the best of our knowledge, our study is the largest conducted in this cohort. Another retrospective study investigated the safety and efficacy of EBUS-TBNA specifically in this category of patients; in this study, 111 patients aged 80 years or older were enrolled; procedures were performed under moderate sedation using midazolam and/or fentanyl; the overall complication rate was 5%, and the diagnostic rate was 75% [19].

Overall, the complication rate observed in our survey was 11.3%; these were mostly minor and transient; the most frequent adverse event was blood pressure elevation requiring the administration of intravenous antihypertensive drugs such as clonidine (4.3%); this finding is similar to other clinical reports. Particularly, Tunc et al. analyzed the safety of EBUS-TBNA under deep sedation in elderly, finding that hypertension was higher in the elderly group with a rate of 14.1%. In this study, they compared patients aged 65 years and older with patients aged 45 years and under; the sedation protocol included the use of propofol alone or in combination with other drugs such as midazolam and ketamine. Hypertension was more frequent in the elderly group in which ketamine was used, suggesting that this group was more sensitive to the effects of ketamine [20].

A prospective cohort study of patients undergoing EBUS-TBNA under moderate sedation, dividing patients into two age categories (<70 years or ≥70 years old), reported no significant difference in overall complications between the younger and the elderly group (8.7 vs. 5.1% *p*: 0.13); nevertheless, it is interesting to note that major complications occurred in the ≥70-year-old category [21].

Another retrospective study of patients undergoing EBUS-TBNA under moderate sedation, dividing patients into two age categories (<65 years or ≥65 years old), reported that the incidence of complications was similar in the two groups (3.9% vs. 4.4% *p*: 0.87); nevertheless, in this study, only 15 patients aged ≥ 80 years old were included [22].

Complication rates reported in the literature vary widely. In a recent review, overall complications from EBUS ranged from 0.04% to 17%, and the most common were infectious complications (0.04–4%) including mediastinitis, pneumonia, pericarditis, bacteriemia or fever, abscess and tumor bed infection; a possible mechanism of infectious complications is the inoculation of oropharyngeal bacteria into the target tissue during the transbronchial passage of the needle; the presence of necrotic or cystic lesions, multiple nodal sampling or patient conditions such as immunosuppression, diabetes or bronchial colonization are risk factors for infectious complications [23]. A large retrospective study by Asano et al., which included 7345 patients, reported a complication rate of 1.23%; hemorrhage was the most frequent complication (0.68%), followed by infectious complications (0.19%) [24].

Complications observed in our study are slightly higher than those reported in the literature. This can be partly explained by a lack of uniform definition of “elderly”. Studies often include younger patients, whereas patients aged ≥ 80 years represent only a small part of the samples; for this reason, our data are difficult to compare with those of other studies in the literature regarding EBUS-TBNA in elderly patients. Kaya et al. categorized patients aged 65 years or older as the elderly group; all EBUS-TBNA procedures were performed under moderate sedation with midazolam and/or fentanyl and overall complications were 5.3% with similar rate recorded both in the elderly and younger groups (5.4 vs. 5.2%, *p* = 0.386). However, a subgroup analysis of about 107 patients aged 80 years and over showed a complication rate of 9.3%, a value closer to the complication rate detected in our study [25]. Other studies divided patients into older (≥70 years) and younger (<70) groups and concluded that EBUS-TBNA is a safe procedure without significant differences between the two groups in overall incidence of complication; nevertheless, in these studies, the sample size of the older group was relatively small [26,27].

Furthermore, the lack of a uniform definition of complication and differences in the duration of follow-up period after EBUS-TBNA for detecting adverse events may partly explain the discrepancy between our complication rates and those reported int the literature. In our study, we meticulously reported any adverse event during the procedure and up to 1-week after it; in the study by Demirci e al. complications were recorded only within 24 h of the procedure [28].

The incidence of major complications was low (1.4%) and similar to that reported in previous studies conducted on the elderly [21]; the only death, which occurred in the week following the EBUS-TBNA, was not associated with the endoscopic procedure, but rather related to the rapid deterioration of general condition with persistent thrombocytopenia, hematemesis with severe anemia, and onset of respiratory failure with CT findings consistent with a lymphangitic pattern.

We did not observe any infectious complications (mediastinitis, abscess formation) in contrast to the literature data reporting them among the most frequent severe adverse events related to EBUS-TBNA [29]; moreover, we did not record any severe bleeding.

In our patients, there was no difference in survival between those who experienced complications and those who did not, and no difference was reported in patients older or younger than 85 years in terms of survival after bronchoscopy. The only factor associated with survival was the presence of a diagnosis of solid tumor (*p* < 0.0001). The occurrence of complications was not associated with age, sex, BMI and CCI at the multivariate logistic regression level.

In most cases, TBNA sampling was performed (99.3%, 140/141); only in one case (0.7%, 1/141) was it necessary to repeat the procedure with anesthesiologic assistance, confirming the literature data about the tolerance of EBUS TBNA under moderate sedation, refs. [9,10,11,12] also in elderly patients, and suggesting that age itself is not enough to plan EBUS-TBNA with anesthesiologic assistance.

The adequacy of specimens was 96.4% (135/140 samples); this finding is consistent with the previous literature studies involving both the general population [30] and the elderly. Specifically, in the study by Evison et al., adequacy was 95% in patients aged ≥ 70 years [21]. In five patients with inadequate sampling, another diagnostic procedure was planned. One patient underwent CT-guided fine-needle aspiration, which led to a diagnosis of NSCLC, but no subsequent therapy was possible because the patient died a few days later. One patient repeated EBUS-TBNA with sampling of another nodal station (2R instead of 4R), achieving a diagnosis of NSCLC. One patient underwent rigid bronchoscopy with sampling of station 4R instead of 10R, showing the presence of anthracosis. One patient underwent medical thoracoscopy, which showed non-specific pleuritis, and radiological follow-up was continued. In the last case, a surgical pleural biopsy was performed, evidencing the presence of an epithelioid mesothelioma, and oncological treatment was undertaken.

Sampling was adequate but without a specific diagnosis in 15 patients (10.7%); in most of these patients, a second diagnostic procedure was not scheduled, so sensitivity and specificity could not be calculated. Subsequent surgical sampling was performed in six of these patients (6/15, 40%), confirming the negative EBUS-TBNA results.

The diagnostic yield was 85.7%, in line with the literature data regarding the general population [30] or other studies on the elderly like the study of Kaya et al., which includes anthracosis among diagnostic samples; the incidence of anthracosis increases with age [25] and this can explain the high prevalence of anthracosis in our study (42/140, 30%).

Niwa et al. reported a 75% diagnostic rate in patients aged 80 and older [19]. In the study by Dhooria et al., the positivity rate of EBUS-TBNA aspirates was lower in elderly (48.8% in patients ≥ 65) than in younger subjects (66.7% in patients < 65). Specifically, in patients aged ≥ 80, the positivity rate was 53.3%; however, the sample size was quite small, including only 15 patients aged ≥ 80 [22].

The malignancy rate was 52.9% of cases (74/140) and specifically, NSCLC was the most common diagnosis (34.3% of patients), followed by SCLC (10% of patients), metastasis (5.7% of patients) and lymphoma (2.85% of patients).

Among oncological patients, 58 subjects (58/74, 78.4%) were treated, highlighting the importance of proceeding with diagnostic investigation, also in elderly patients, in order not to preclude a therapeutic proposal. On the other hand, it is noteworthy that eight patients (8/141, 5.7%) died within one month after the bronchoscopy; for all of them, an oncological diagnosis was made; the deaths were not related to the endoscopic procedure but due to a rapid deterioration of clinical condition in the following days due to the progression of the underlying disease. However, this finding underlines the importance of an accurate selection of patients undergoing EBUS-TBNA and, more generally, diagnostic procedures, carefully balancing risks and benefits, also considering performance status and life expectancy.

The present study has some limitations. First, it is a retrospective analysis and selection bias cannot be excluded, as patients deemed unfit for the procedure may have been excluded. Second, the study was conducted at a single center, and the sample size was relatively small. Another limitation is that, when a specific diagnosis was not reached, a second procedure was not always performed to confirm the results. Furthermore, this retrospective study does not include a control group consisting of younger patients who underwent the same procedure or of patients of the same age who underwent the procedure under a different form of sedation, and this can limit the strength of our conclusions.

## 5. Conclusions

The results of this study suggest that EBUS-TBNA under moderate sedation can be considered a safe and effective procedure even in patients over 80 years of age, but a careful assessment of the patient’s general condition and life expectancy, with appropriate balancing of risks and benefits, is recommended before performing the procedure.

Elderly patients can benefit from the procedure by obtaining a diagnosis and, potentially, subsequent therapeutic options.

Clinicians performing EBUS-TBNA should be aware of types of possible complications that can occur during the procedure to ensure timely intervention.

## Figures and Tables

**Table 1:** Patient characteristics.

Number of patients	141
Age years, mean ± SD	82.6 ± 2.2
Sex	
Male, *n* (%)	84 (59.6)
Female, *n* (%)	57 (40.4)
Body Mass Index, mean ± SD	25.7 ± 3.8
Charlson Comorbidity Index, mean ± SD	5.2 ± 0.9
ASA, *n* (%)	
1	9 (6.4)
2	45 (31.9)
3	87 (61.7)

**Table 2:** Sampling of lymph nodes according to IASLC classification or lesions adjacent to central airway during EBUS-TBNA in patients aged ≥ 80 years.

	Total (*N*: 141)
Sampling not performed *n* (%)	1 (0.7%)
Sampling performed *n* (%)	140 (99.3%)
2L	4
2R	5
4R	60
4L	27
7	65
10L	4
10R	10
11L	22
11R	33
12R	1
Lesions adjacent to central airway	13
Multiple sampling sites	65 (46.1%)

**Table 3:** Sedation during EBUS-TBNA in patients aged ≥ 80 years.

Sedation	Dose (mg)
Midazolam (mg), mean ± SD	4.6 ± 2
Fentanyl (mg), mean ± SD	0.1 ± 0.09
Meperidine (mg), mean ± SD	72.4 ± 12.4

**Table 4:** EBUS-TBNA outcomes in patients aged ≥ 80 years.

	Total (*N*: 140)
**Adequate**	**135 (96.4%)**
Adequate without a specific diagnosis	15 (10.7%)
Adequate with a specific diagnosis	120 (85.7%)
Malignancy	74 (52.9%)
Non-small cell lung cancer	48 (34.3%)
Small cell lung cancer	14 (10%)
Metastatic malignancy	8 (5.7%)
Lymphoma	4 (2.85%)
Benign Pathology	46 (32.8%)
Anthracosis	42 (30%)
Granulomatous disorders	4 (2.85%)
**Inadequate**	**5 (3.6%)**

**Table 5:** Treatment outcome in patients aged ≥ 80 years subsequently diagnosed with malignancy.

	Total (*n*: 74)
**Active treatment**	**58 (78.4%)**
Systemic oncological therapy	29 (39.2%)
Radiotherapy	20 (27%)
Radiotherapy and systemic oncological therapy	9 (12.2%)
**Best supportive care**	**16 (21.6%)**

**Table 6:** Complications of EBUS-TBNA in patients aged ≥ 80 years.

	Total (*N*: 141)
All	16 (11.3%)
**Major**	**2 (1.4%)**
Pneumothorax	1 (0.7%)
Epistaxis with hospital admission	1 (0.7%)
**Minor**	**14 (9.9%)**
Blood pressure elevation	6 (4.3%)
Hypoxemia	3 (2.1%)
Mild epistaxis	2 (1.4%)
Laryngospasm	1 (0.7%)
Minor bleeding	1 (0.7%)
Cough with poor tolerance to the procedure	1 (0.7%)

**Table 7:** Time to death after EBUS-TBNA in patients aged ≥ 80 years.

	Total (*n*: 141)
Death within one months following EBUS-TBNA	8 (5.7%)
Death within three months following EBUS-TBNA	18 (12.8%)

## Data Availability

The data presented in this study are available upon request from the corresponding authors. The data are not publicly available due to the privacy policy for clinical information in Italy.

## Abbreviations

The following abbreviations are used in this manuscript:

EBUS-TBNA	Endobronchial ultrasound guided transbronchial needle aspiration
CT	Computed tomography
FDG-PET	Fluorodeoxyglucose position emission tomography
IV	Intravenous
ROSE	Rapid on-site evaluation
BMI	Body Mass Index
CCI	Charlson Comorbidity Index
ASA	American Society of Anesthesiologists
IASLC	International Association for the Study of Lung Cancer
NSCLC	Non-small cell lung cancer
SCLC	Small cell lung cancer
